# A necroptosis -related signature for predicting prognosis and immunotherapy in hepatocellular carcinoma

**DOI:** 10.3389/fgene.2022.919599

**Published:** 2022-09-05

**Authors:** Xing Fu, Yuling Yang, Xiaozhi Zhang

**Affiliations:** ^1^ Department of Radiation Oncology, Ankang central hospital, An’kang, Shaanxi Province, China; ^2^ Xi’an Medical University, Xi’an, China

**Keywords:** hepatocellular carcinoma, prognosis, immunotherapy, TCGA, necroptosis

## Abstract

**Background:** Hepatocellular Carcinoma (HCC) is an aggressive tumor with an inferior prognosis. Necroptosis is a new form of programmed death that plays a dual effect on the tumor. However, the role of necroptosis-related genes(NRGs) in HCC remains unknown.

**Methods:** All datasets were downloaded from publicly available databases. The consensus clustering analysis was used to classify patients into different subtypes based on NRGs. The Least Absolute Shrinkage and Selection Operator (LASSO) Cox regression were used to develop a prognostic signature. Tumor Immune Dysfunction and Exclusion (TIDE) was used to predict immunotherapy response.

**Results:** The genetic and transcriptional changes of NRGs were observed in HCC. Patients were classified into three clusters based on differentially expressed NRGs, of which Cluster-3 had the worst prognosis and the highest immune infiltration. The prognostic signature was developed based on 8-NRGs, which have shown excellent prognostic performance. The high-risk group in the signature presented significantly higher immune infiltration, such as aDCs, iDCs, macrophages, and Treg, compared to the low-risk group. TMB and immune checkpoints were also higher in the high-risk group. Moreover, a lower TIDE score was observed in the high-risk group, indicating the patients with high risk-score may be suitable for immunotherapy. *Via* the dataset of IMvigor210, we found a higher risk score in the immunotherapy response group.

**Conclusion:** We developed a new necroptosis-related signature for predicting prognosis with the potential to predict immunotherapy for HCC patients.

## Introduction

Liver cancer is the sixth most frequent cancer and the third leading cause of cancer-related deaths globally, posing a massive threat to public health (Sung et al., 2021). The most common histologic subtype of liver cancer is hepatocellular carcinoma (HCC), which accounts for approximately 90% of all cases (Llovet et al., 2021). The major risk factors for HCC are chronic hepatitis virus (HBV and HCV), heavy alcohol consumption, and non-alcoholic fatty liver disease (Llovet et al., 2021). Despite the great benefits of evolving treatments for HCC (Villanueva, 2019), it remains one of the tumors with the poorest prognosis (Altekruse et al., 2014).

Necroptosis is a new form of programmed cell death mediated by RIP1, RIP3, and MLKL in a caspase-independent way (Khan et al., 2021). Necroptosis and apoptosis have the same upstream molecular machinery (Khoury et al., 2020), while the morphology and immunology are distinctly different (Bertheloot et al., 2021). Plasma membrane rupture of necrotic cells releases cellular contents, exposing injury-related molecular patterns that induce inflammatory responses (Khan et al., 2021). The contents of apoptotic cells are encapsulated by apoptotic bodies further engulfed by neutrophils, macrophages, or dendritic cells for final degradation (Wong, 2011), thus having lower immunogenicity. Caspase-8 determines the type of cell death, with activation of Caspase-8, promotes apoptosis, while inhibition of Caspase-8 activity shifts the balance to necroptosis (Newton et al., 2019). Many studies have demonstrated that necroptosis has a vital role in tumorigenesis, metastasis, and tumor microenvironment (TME) (Stoll et al., 2017; Seehawer et al., 2018). However, there are few studies on necroptosis-related genes (NRGs) in patients with HCC. Therefore, it is necessary to explore the role of NRGs in HCC.

This study systematically investigated the expression, somatic mutation, and copy number variation of NRGs in HCC. We also developed a prognosis signature based on NRGs, correlated with immunotherapy.

## Materials and methods

### Acquisition of gene expression and clinical data

Level-3 RNA sequencing data (normalized FPKM), somatic mutations (VarScan2), copy number variation (CNV), and clinical data of HCC were obtained from the TCGA (The Cancer Gene Atlas). Samples with incomplete clinical information or survival time less than 30 days were not included. From the ICGC (International Cancer Genome Consortium), RNA sequencing data of 243 HCC samples and clinical information were obtained. The datasets of IMvigor210 (http://research-pub.gene.com/IMvigor210CoreBiology) include transcriptomic and clinical data of 348 patients with metastatic uroepithelial carcinoma treated with anti-PDL-1. All RNA sequencing data were log2 transformed for further analysis.

GSE14520 and GSE54236 were obtained from Gene Expression Omnibus (GEO) (https://www.ncbi.nlm.nih.gov/geo/). From GSE14520 and GSE54236, we can get gene expression and survival information for 220 and 82 HCC patients, respectively. GEO data probes use the provided annotation files to match gene symbols. When multiple probes matched a gene, the average value was taken.

### The landscape of necroptosis-related genes

A total of 159 necroptosis-related genes (NRGs) were obtained from the Kyoto Encyclopedia of Genes and Genomes (KEGG: https://www.kegg.jp/). Somatic mutations were visualized using the “maftools” R package (Mayakonda et al., 2018). The CNV frequency of NRGs was visualized using the “ggplot” R package. Differentially expressed NRGs (DE-NRGs) between HCC and normal samples were screened using the Wilcox test, with |Log2FC|>1 and FDR *p*-value < 0.05.

### Consensus clustering analysis

Consensus clustering analysis was performed based on DE-NRGs by using the “ConsesusClusterPlus” R package (1,000 iterations and resample rate of 80%) (Wilkerson and Hayes, 2010). The samples were divided into k (ranging from 2 to 10) groups by the k-means algorithm. The consensus distribution for each k was shown by the cumulative distribution function (CDF) plot. The CDF plot facilitated finding k with an approximate maximum distribution, which indicates maximum stability. The average consensus value between an item and the cluster members was measured by item consensus (IC). The average pairwise IC of items in a consensus cluster was estimated by cluster consensus (CLC). The Kaplan-Meier curve was used to assess survival differences among subtypes. Stromal-Score, Immune-Score, and ESTIMATE-Score were calculated by using the “estimate” R package (Yoshihara et al., 2013). The abundance of two stromal cells and eight immune cells were calculated by using the “MCP-counter” R package (Becht et al., 2016).

### Construction of the prognostic signature

The univariate Cox regression analysis was performed to screen prognostic genes. The Cox proportional hazards model (iteration = 1,000) with the Least Absolute Shrinkage and Selection Operator (LASSO) penalty on the prognostic genes to develop necroptosis-related signature by using the “glmnet” R package. The risk score is equal to the sum of the expression of each gene multiplied by the corresponding coefficient. The patients were divided into high- and low-risk groups according to the median risk score in the TCGA and ICGC cohorts.

### Verification of the prognostic signature

Time-dependent receiver operating characteristic (ROC) curve analysis was performed by using the “Survival” and “ROC” R packages. Kaplan-Meier survival curves were applied using the “survival” and “survminer” R packages. The database of Human Protein Atlas (HPA: https://www.proteinatlas.org/) was used to explore the expression of the protein encoded by NRGs between HCC and normal samples (Uhlen et al., 2017).

### Independent prognosis of risk signature and nomogram construction

The multivariate Cox regression analysis was used to assess the independence of risk signature. The risk score and other parameters were used to construct a nomogram to predict individual survival using the “rms” R package. The calibration curve was used to measure the accuracy of the predictions.

### Functional enrichment analysis

The DEGs between the high- and low-risk groups in the TCGA cohort were screened by the Wilcox test, with |Log2FC|>1 and FDR *p*-value < 0.05. Metascape is a convenient web application for pathway and functional enrichment analysis (Zhou et al., 2019). Put DEGs into the website (https://metascape.org) for enrichment analyses. Gene Set Enrichment Analysis (GSEA) was performed to identify hallmark gene sets enriched in the high-risk group (Liberzon et al., 2015), with normal *p*-value < 0.05 and FDR *p*-value <0.25.

### Somatic mutations and TMB in the risk signature

The “maftools” package was applied to visualize HCC mutation data. The function “tmb” in the R package " mafools “was applied to calculate the value of TMB from mutation data. The function “surv_cutpoint” in the R package “survminer” was applied to select the optimal cut-off value of TMB and divided patients into high-TMB and low-TMB group.

### Immune status in the risk signature

The enrichment scores of 16 types of immune cells and 13 immune-related pathways were calculated *via* single-sample gene set enrichment analysis (ssGSEA) by using the “GSVA” and “GSEABase” R package. Tumor Immune Dysfunction and Exclusion (TIDE) web application (http://tide.dfci.harvard.edu) was used to predict immunotherapy response (Fu et al., 2020). The higher TIDE score, the worse immunotherapy response might be.

### Drug sensitivity analysis

The“pRRophetic” R package based on Genomics of Drug Sensitivity in Cancer (www.cancerrxgene.org/) was used to calculate the half-maximal inhibitory concentration (IC50) of drugs (Geeleher et al., 2014).

### Statistical analysis

**FIGURE 1:**
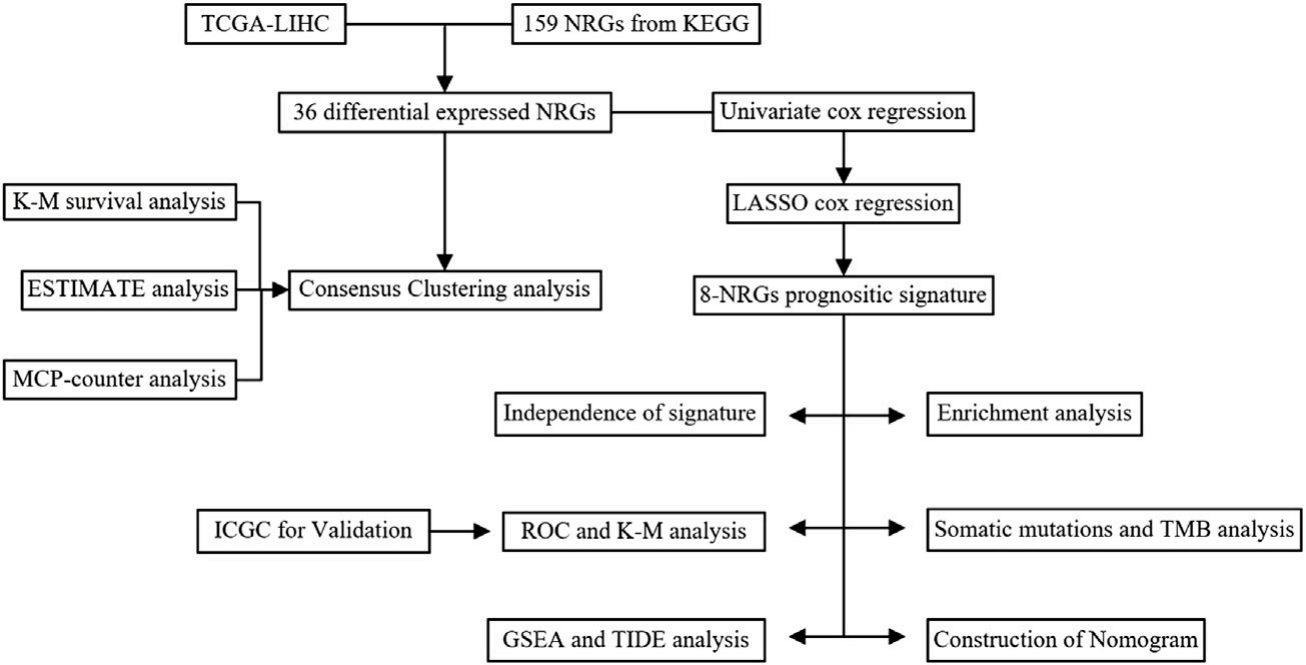
The flowchart of this study.

All statistical analyses were performed using R software (version 4.1.2). *p*-value <0.05 is significant if not specified. The study flowchart is shown in (Figure 1).

## Results

### The landscape of Genetic Variation of NRGs

**FIGURE 2:**
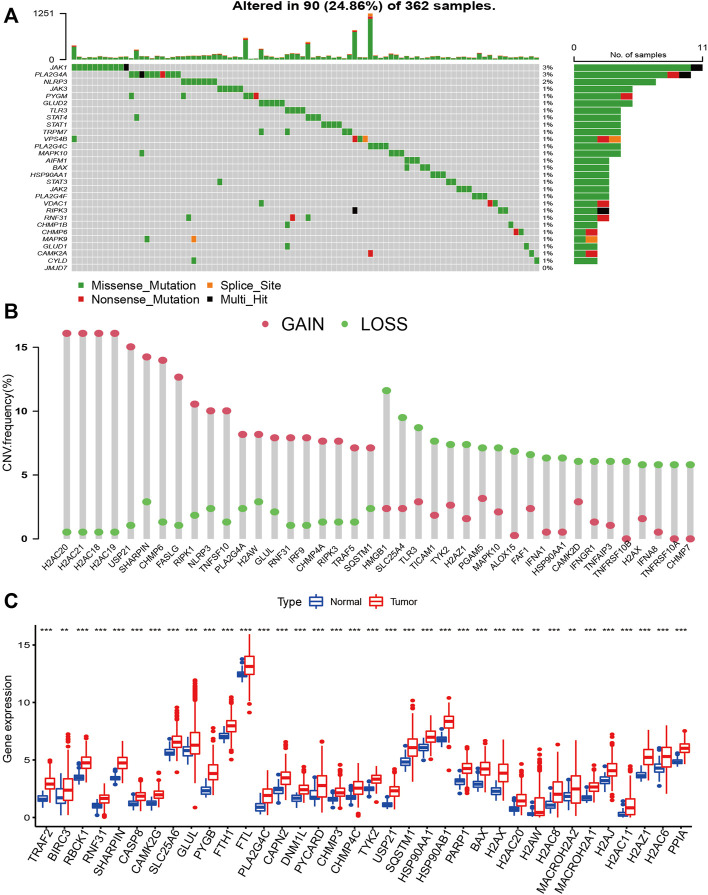
The landscape of Genetic Variation of NRGs. **(A)** The waterfall plot shows the top 30 NRGs with high mutation frequency in HCC patients; **(B)** The bar chart shows the frequency of the top 20 gain alteration and loss alteration of NRGs; **(C)** the Bar plot shows different expressed NRGs between HCC and normal samples. (*p < 0.05; **p < 0.01; ***p < 0.001).

The waterfall plot shows the top 30 NRGs with high mutation frequency in HCC patients (Figure 2A). The frequencies of NRGs mutations were low, and most samples were missense mutations. CNV of NRGs was common in HCC, most of which were gain alterations (Supplementary Table S1). The bar chart shows the top 20 NRGs with the high frequency of gain and loss alteration (Figure 2B). The top five genes with the highest frequencies of gain alterations were H2AC20, H2AC21, H2AC18, H2AC19, and USP21. In contrast, HMGB1, SLC25A4, TLR3, TICAM1, and TYK2 had the highest frequency of loss alterations. A total of 36 differentially expressed NRGs were identified between HCC and normal samples, and all these genes were upregulated in HCC samples (Figure 2C).

### Consensus clustering analysis

**FIGURE 3:**
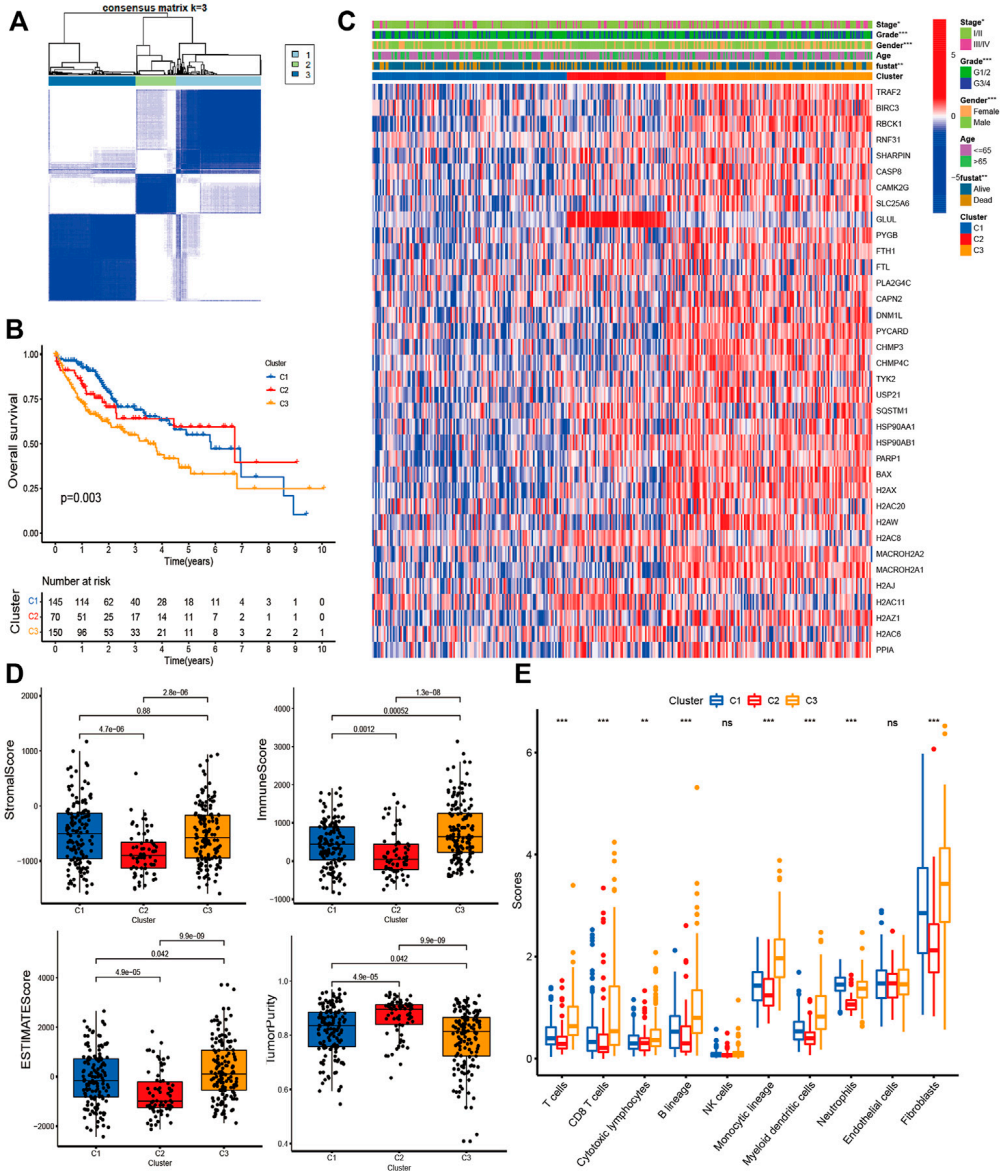
Consensus Clustering analysis. **(A)** Consensus matrix when the number of clusters (k) = 3; **(B)** Kaplan-Meier curves show the survival differences between subtypes; **(C)** The heatmap shows the expression of NRGs and clinicopathological features between subtypes; **(D)** Bar plot shows the Stromal score, Immune score, ESTIMATE score, and Tumor purity between subtypes; **(E)** Bar plot shows the immune inflation between subtypes.

To explore the immunological and prognostic implications, we attempted to classify HCC into different subtypes based on DE-NRGs. The CDF plot shows a slight increase in CDF but a greater decrease in CLC when k was equal to or greater than 4, and when *k* = 3, the CDF was higher than *k* = 2 (Supplementary Figure S1). Therefore, we divided the HCC patients into three clusters. The consensus matrix indicated the greatest correlation within-subtype and difference among subtypes when *k* = 3, also indicating the practicability of dividing the patients into three clusters (Figure 3A). The Kaplan-Meier curve revealed significant survival differences between subtypes (*p* < 0.01), among which Cluster-3 had the worst survival (Figure 3B). The heatmap revealed the expression of NRGs and clinicopathological features between subtypes (Figure 3C). The distribution of stage, grade, gender, and survival status were different between subtypes. We also found significant differences in immune status among subtypes. The Stromal-score and ESTIMATE-score were highest in Cluster-3, and conversely, it had the lowest tumor purity (Figure 3D). Most immune cells such as T cells, CD8 T cells, cytotoxic lymphocytes, and monocyte lineages were significantly infiltrated in Cluster-3 (Figure 3E).

### Construction of risk signature and validation

We performed a univariate Cox analysis to explore the prognostic value of DE-NRGs in HCC. The forest plot shows that 19 NRGs were associated with survival (Supplementary Figure S2). Next, we developed a risk signature based on 8 NRGs (USP21, H2AZ1, H2AX, CHMP3, PPIA, SQSTM1, DNM1L, and HSP90AA1) using LASSO Cox regression analysis (Supplementary Figure S2). The correlation between the eight genes was significant (Supplementary Figure S2). The following formula quantified the risk score: risk score = [0.13029175×mRNA expression level of USP21] + [0.115665765×mRNA expression level of H2AZ1] + [0.058682133×mRNA expression level of H2AX]+ [0.006653262×mRNA expression level of CHMP3]+ [0.197273978×mRNA expression level of PPIA]+ [0.16888285×mRNA expression level of SQSTM1]+ [0.027048508×mRNA expression level of DNM1L]+ [0.184836598×mRNA expression level of HSP90AA1].

**FIGURE 4:**
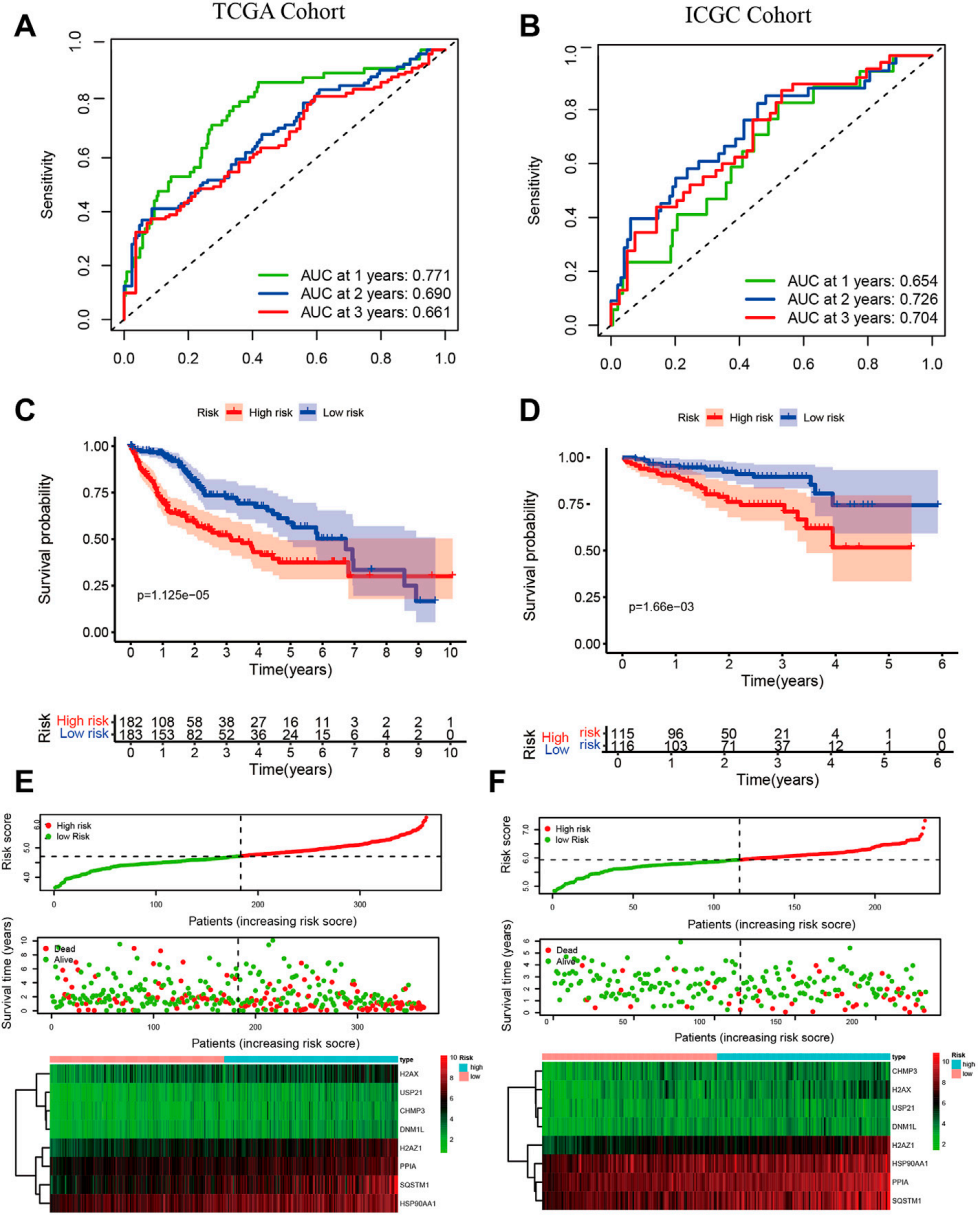
Validation of risk signature. Time-dependent ROC for predicting 1-, 2-, and 3-years OS in the TCGA cohort **(A)** and the ICGC cohort **(B)**; Kaplan-Meier survival curves show different OS between high- and low-group in TCGA **(C)** and ICGC **(D)**; The risk score, survival status, and the heatmap of 8 NRGs between high- and low-group in TCGA **(E)** and ICGC **(F)**. Independent prognostic indicator and nomogram construction.

To assess the prognostic performance of the risk signature, we applied a time-dependent ROC curve analysis. The area under ROC curves (AUCs) for prediction of the 1-, 2-, and 3-years survival rates were 0.771, 0.690, and 0.661 in the TCGA cohort (Figure 4A), and 0.654, 0.726, and 0.704 in the ICGC cohort (Figure 4B). Kaplan-Meier survival curves indicated a poorer OS for the high-risk group compared to the low-risk group in both cohorts. (*p* < 0.01 Figures 4C,D). The distribution of risk score, survival status, and the heatmap of 8 NRGs are summarized in (Figures 4E,F). The 8 NRGs were differentially expressed between high and low-risk groups, and there were more deaths in the high-risk group. Excellent prognostic performance of risk signature was also found in the GSE14520 and the GSE54236 cohort (Supplementary Figure S3).

**FIGURE 5:**
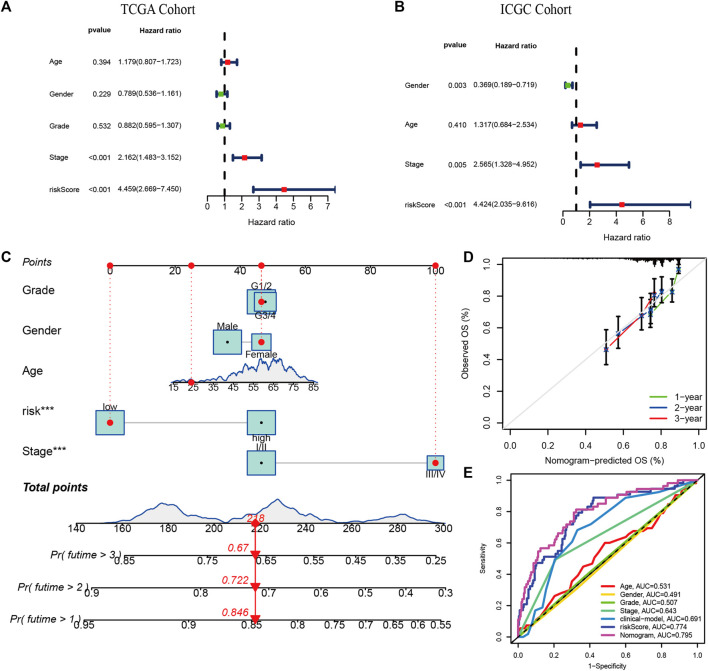
Independent prognostic value of risk signature and Nomogram construction. The multivariate Cox regression analysis in the TCGA cohort **(A)** and the ICGC cohort **(B)**; **(C)** The nomogram for predicting 1-, 2-, and 3-years OS in the TCGA cohort. **(E)** The calibration plots. **(D)** Multi-indicator ROC curves for predicting 1-year OS in the TCGA cohort.

We applied multivariate Cox regression analysis to explore the independent prognostic value of the risk signature. The risk score and stage were independent prognostic indicators in both cohorts (*p* < 0.001, Figures 5A,B). The risk score, age, gender, grade, and stage were used to develop a Nomogram for predicting 1-, 2-, and 3-years OS (Figure 5C). The estimated and actual survival probabilities were well matched, as shown in the calibration curves (Figure 5D). A clinical model was constructed by using traditional clinicopathological parameters.The risk score had higher AUC values than clinical model, indicating that the risk score outperformed these clinicopathological parameters in predicting OS of HCC (Figure 5E).

### Validation of the expression of NRGs in signature

The expression of 8-NRGS in the signature were verified in the ICGC cohort. As shown in (Supplementary Figure S4), 8-NRGS were upregulated in HCC samples (*p* < 0.001). The proteins encoded by 8 NRGs were upregulated in HCC tissues by the database of HPA (Supplementary Figure S4).

### Functional enrichment analysis

We also performed the enrichment analysis based on differentially expressed genes between the high- and low-risk groups to elucidate the biological functions and pathways associated with risk-score. The bar chart shows the top 20 enriched items of DEGs, such as mitotic cell cycle, adaptive immune response, Cell Cycle, Mitotic, positive regulation of leukocyte activation, and regulation of cell adhesion (Supplementary Figure S5). There are 28 hallmark gene sets enriched in the high-risk (Supplementary Table S2), for example, MTORC signal, DNA repair, PI3K AKT MTOR signal, E2F targets, MYC targets, and G2m checkpoint, P53 pathway, Notch signal, and inflammatory response, Hypoxia (Supplementary Figure S5).

### Comparison of somatic mutations and TMB in the signature

**FIGURE 6:**
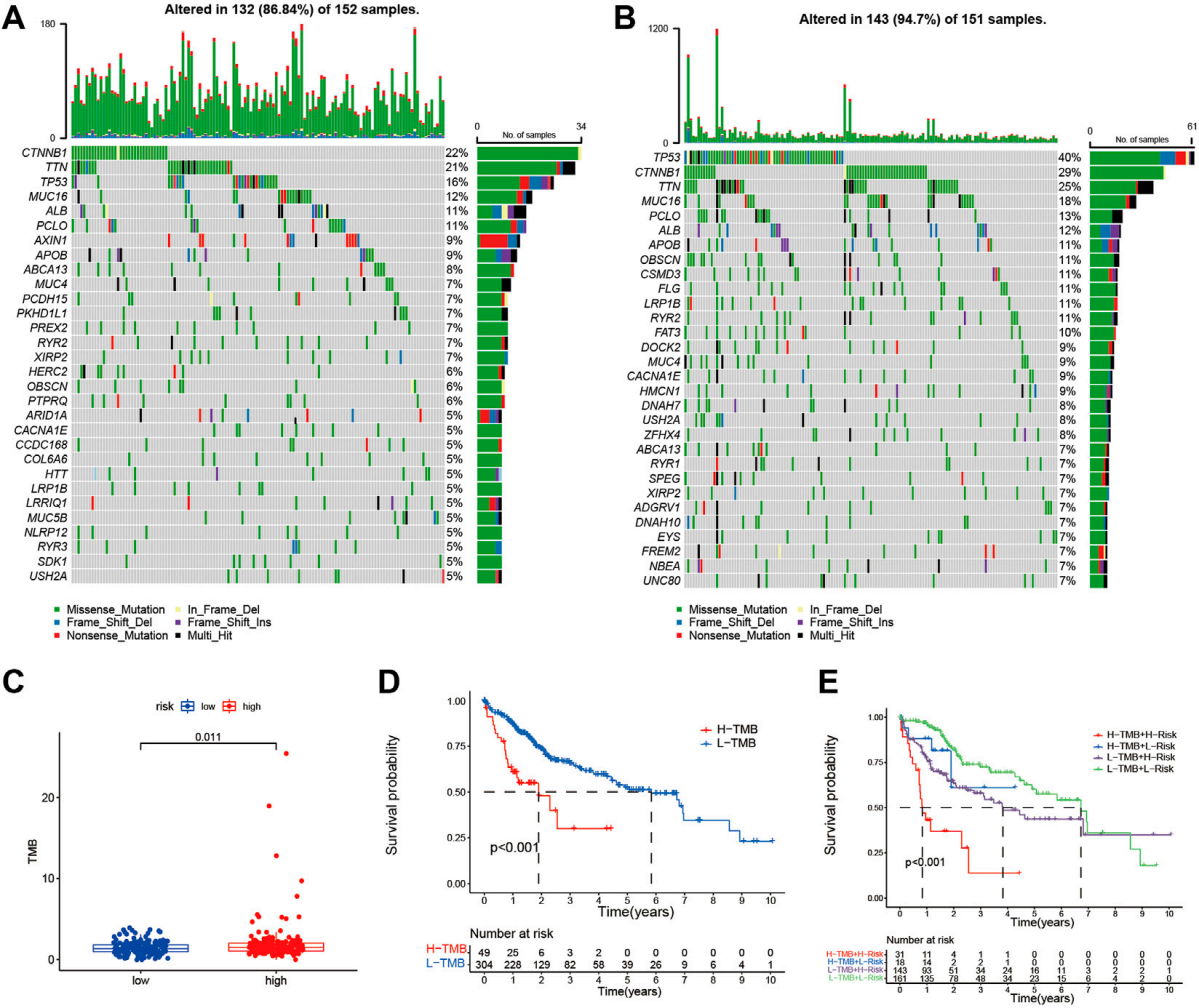
The somatic mutations and TMB in risk signature. The waterfall plot shows the top 30 genes with high mutation frequency in the high-risk group **(A)** and low-risk group **(B)** in the TCGA cohort. **(C)** The bar plot shows the TMB between the high- and low-risk group in the TCGA cohort. **(D)** Kaplan-Meier curve analysis shows survival difference between high and low TMB groups. **(E)** Kaplan-Meier curve analysis showed survival differences among patients classified according to TMB and risk group in the TCGA cohort.

We investigated the differences in somatic mutations between the high- and low-risk groups. TP53 (39%), CTNNB1 (30%), TTN (24%), MUC16 (17%), and ALB (12%) were the top 5 genes with the highest mutation frequency in the high-risk group (Figure 6A), while TNN (22%), CTNNB1 (21%), TP53 (16%), MUC16 (12%), and ALB (10%) were the top 5 genes in the low-risk group (Figure 6B). TMB was significantly higher in the high-risk group than in the low-risk group (*p* < 0.05 Figure 6C). The optimal cut-off was selected and divided patients into a high TMB group and a low TMB group. Patients with low TMB have better survival than those with high TMB (Figure 6D). Patients with low TMB in the low-risk group had the best survival rates, and those with high TMB in the high-risk group had the worst survival rates (Figure 6E).

### Immune activity and prediction of immunotherapy

**FIGURE 7:**
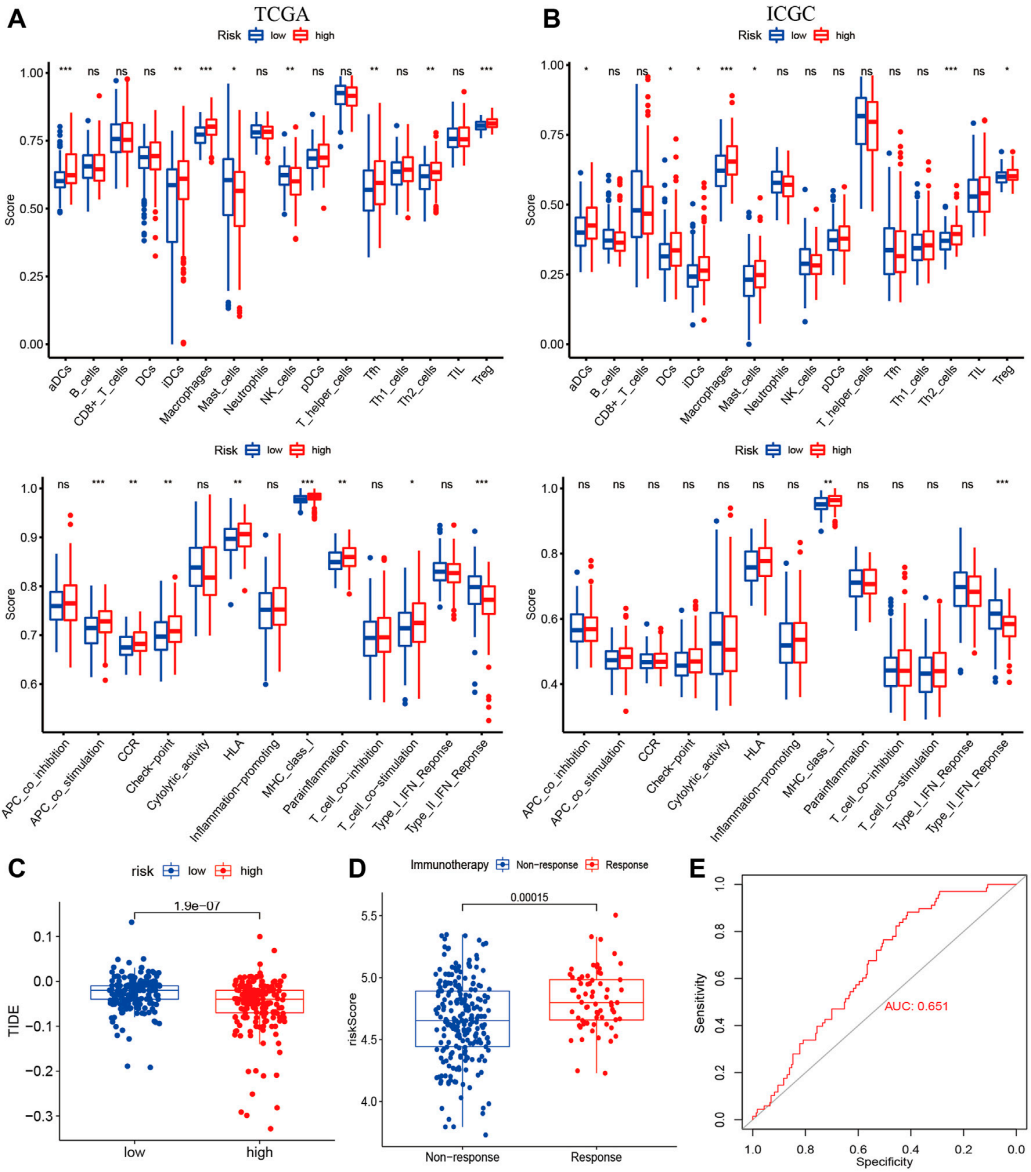
Immune Status and Prediction of response to immunotherapy. The enrichment scores of 16 immune cells and 13 immune-related pathways between subtypes in the TCGA **(A)** and ICGC cohorts **(B)**; **(C)** The TID scores between high- and low-risk groups in the TCGA cohort; **(D)** The risk score between immunotherapy response group (PR/CR) and non-response group (PD/SD) in IMvigor210; **(E)** ROC analysis for predicting immunotherapy response.

To examine the correlation between risk signature and Immune activity, we quantified enrichment scores of 16 types of immune cells and 13 immune-related pathways. The infiltration of aDCs, iDCs, Macrophages, and Treg were higher in the high-risk group in the TCGA and the ICGC cohorts (Figures 7A,B). At the same time, the immune pathway of MHC class-I was active in the high-risk group in both cohorts (Figures 7A,B). Most immune checkpoints were higher in the high-risk group than in the low-risk group in both cohorts (Supplementary Figure S6). TIDE analysis is considered a sound predictor of response to immunotherapy. The TIDE score was lower in the high-risk group (Figure 7C), indicating high-risk patients may be favorable for immunotherapy. We calculated the risk score using the same formula as the TCGA cohort for each patient in IMvigor210. As shown in (Figure 7D), the immunotherapy response group has higher risk score than the non-response group. The AUC for predicting the immunotherapy response was 0.651 (Figure 7E).

### Drug sensitivity analysis in the risk signature

**FIGURE 8:**
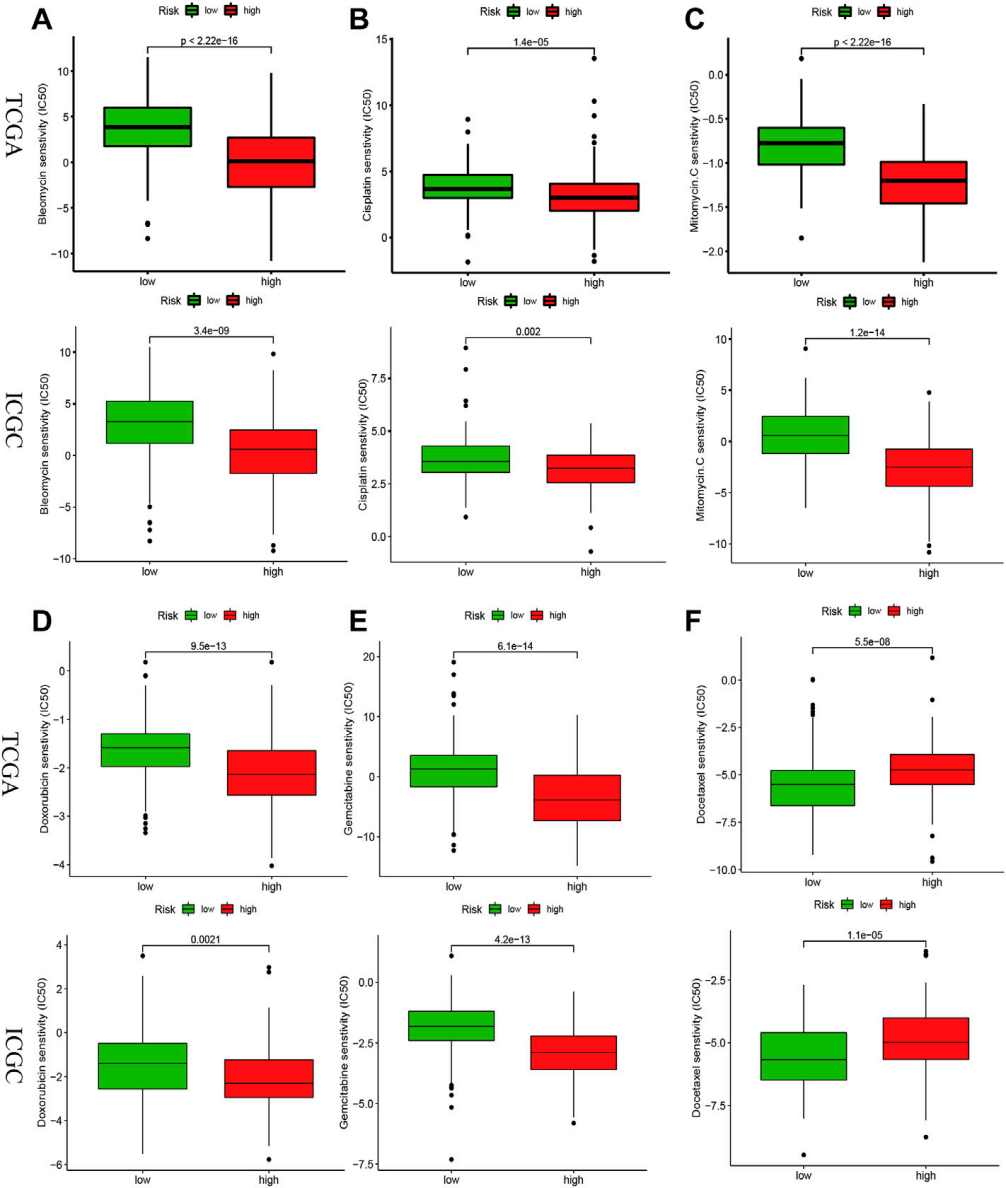
Drug sensitivity analysis in the risk signature. IC50 values for chemotherapeutic agents commonly used for HCC between high- and low-risk groups in the TCGA and ICGC cohorts. Bleomycin **(A)**, Cisplatin **(B)**, MitomycinC **(C)**, Doxorubicin **(D)**, Gemcitabine **(E)**, Docetaxel **(F)**.

We investigated whether risk-score could predict patients’ sensitivity to chemotherapy. We selected chemotherapeutic agents commonly used in HCC. We found that the high-risk group may be more sensitive to Bleomycin, Cisplatin, MitomycinC, Doxorubicin, and Gemcitabine (Figures 8A–E), while Docetaxel for the low-risk group (Figure 8F) in the TCGA and ICGC cohorts, implicating the signature as a prospective predictor of chemotherapy sensitivity.

## Discussion

Cell death plays a critical role in tumorigenesis, development, and treatment and has become a hot topic of research, such as pyroptosis, ferroptosis, and necroptosis (Strasser and Vaux, 2020). The role of necroptosis in tumors is a double-edged sword; as a form of programmed cell death, necroptosis can inhibit tumorigenesis, yet it can trigger inflammatory responses that promote cancer metastasis and immunosuppression (Gong et al., 2019). This study systematically investigated the prognostic and immunological value of NRGs in HCC and may help provide an essential foundation for future research.

The current staging of HCC mainly includes TNM staging, American Joint Committee on Cancer, Barcelona Clinic Liver Cancer, Okuda, Japan Integrated Staging, etc. (Sirivatanauksorn and Tovikkai, 2011). Due to the heterogeneity of tumors, no system can always effectively predict the prognosis and treatment of HCC (Nault et al., 2020). With advances in microarray and RNA sequencing technologies, more and more studies focus on molecular subtypes (Zhu et al., 2022). In this study, HCC patients were classified into three subtypes based on DE-NRGs. Survival rates and immune inflation differed significantly among subtypes, and interestingly, Cluster-3 had the worst OS and the highest immune inflation rate. We also developed an NRG-related signature to predict OS in HCC, which is better than clinicopathology. Compared with NRGs related signatures for HCC which have been published (Li et al., 2022; Yang and Jiang, 2022), our signature had a better prognostic performance (Supplementary Figure S7).

Most NRGs presented in our signature were associated with tumorigenesis and proliferation of HCC. USP21, as an efficient deubiquitylates, promotes the stability of BRCA2 to regulate DNA repair in HCC cell (Liu et al., 2017). USP21 can also promote HCC cell proliferation by activating the ERK signal pathway (Li et al., 2018). H2AZ1 was overexpressed in HCC cell and was associated with a poor prognosis (Yang et al., 2016). H2AZ1 promotes HCC progression by regulating the cell cycle and epithelial-mesenchymal transition (Yang et al., 2016). H2AX is a biomarker for DNA double-strand breaks, which can facilitate base excision repair (Chen et al., 2021). *γ* -H2AX was elevated in precancerous lesions of HCC, suggesting an essential role in the development of HCC (Matsuda et al., 2013). *γ*-H2AX promotes HCC angiogenesis *via* the EGFR/HIF-1α/VEGF pathway (Xiao et al., 2015). CHMP3 is a component of the ESCRT-III protein complex (Solomons et al., 2011). ESCRT III repairs nuclear envelope rupture during cell migration, limiting DNA damage and cell death (Raab et al., 2016). PPIA belongs to the immunophilin family and is involved in the anti-apoptotic cancer cell (Daneri-Becerra et al., 2021). PPIA promotes HCC cell metastasis by regulating MMP3 and MMP9(33). SQSTM1 is an autophagy-associated protein that has a crucial role in controlling cell death and survival (Moscat and Diaz-Meco, 2009). SQSTM1 promotes HCC development through activation of NRF2 and mTORC1, induction of c-Myc, and resistance to oxidative stress (Umemura et al., 2016). Activating the SQSTM1/p62-Keap1-NRF2 pathway can inhibit ferroptosis in HCC cells (Sun et al., 2016). DNM1L is critical for regulating mitochondrial fission and cell proliferation, preventing calcium-mediated apoptosis (Chan, 2020). DNM1L promotes the expansion of mitochondrial division in HCC cells by mediating the p53/p21 and NF-κB/cyclins pathways (Zhan et al., 2016). HSP90AA1 regulates MLKL oligomerization and plasma membrane translocation and is required for TNF-induced necroptosis (Zhao et al., 2016). HSP90AA1 promotes HCC invasion by promoting epithelial-mesenchymal transition and inhibiting tumor stem cell apoptosis (Gao et al., 2015).

To explore the differences in biological functions and pathways between high and low-risk groups, we performed GSEA analysis. More than half of hallmark gene sets (28/50) were enriched in the high-risk group (Supplementary Table S2), and these pathways were involved in tumor progression and metastasis (Liberzon et al., 2015). Notably, these pathways were also associated with antitumor immunity, such as DNA repair (Mouw et al., 2017), P53 pathway (Mantovani et al., 2019), Notch signal (Yuan et al., 2010), Inflammatory response (Ozga et al., 2021), Wnt beta-catenin signal (Luke et al., 2019), PI3k Akt mTOR signal (O'Donnell et al., 2018). Moreover, we found a higher frequency of TP53 mutations in the high-risk group (39 *vs*. 16%). TP53 is a key fail-safe mechanism of cellular anti-cancer defenses, and TP53 mutation was strongly associated with tumorigenesis, progression, and antitumor immunity (Donehower et al., 2019). Thus, enrichment and mutation analysis can partially explain the survival and immune status differences between the high-risk and low-risk groups.

Then, the correlation between risk score and cancer immune status was investigated. The infiltration of aDCs, iDCs, Macrophages, and Treg in the high-risk group (Figures 7A,B), which were involved in shaping TME. Dendritic cells, as one of the most important regulators of adaptive immune responses, are crucial for T-cell-mediated cancer immunity (Gardner and Ruffell, 2016). M1 macrophages can induce PD-L1 expression through the IL-1β signal in HCC (Zong et al., 2019). M2 macrophages can promote the development of HCC, and metastasis and suppress anti-tumor responses through multiple pathways (Tian et al., 2019). Treg cells can enhance tumor progression and repress antitumor immune responses, and the usage of anti-CTLA-4 antibodies can effectively kill effector Treg cells (Tanaka and Sakaguchi, 2017). The immune pathway of MHC class-I was active in the high-risk group, which was essential in regulating innate and adaptive cytotoxic responses (Dhatchinamoorthy et al., 2021). The TMB and immune checkpoints were higher in the high-risk group, commonly employed as immunotherapy predictors (Chan et al., 2019; Morita et al., 2021).

Immune checkpoint inhibitors, a remarkable breakthrough in oncology treatment, have brought tremendous benefits to patients with malignant tumors (Szeto and Finley, 2019). Checkmate-040 and KEYNOTE-240 trials revealed the great potential of immunotherapy in HCC (El-Khoueiry et al., 2017), (Finn et al., 2020). Necroptosis is a form of inflammatory cell death that regulates antitumor immunity (Gong et al., 2019). Therefore, HCC patients with different necroptosis features and immune statuses may have different outcomes to immunotherapy.

TIDE, an algorithm for predicting immune response, was lower in the high-risk group, indicating a better immune response in high-risk score patients. In addition, the higher risk score was associated with the immunotherapy response group by datasets of IMvigor210.

We used retrospective data from public databases to construct and validate. Thus, more prospective real-world data are required to validate their clinical value. In addition, our study lacks validation by *in vivo* and *in vitro* experiments. Therefore, the next step should be to validate the function of NRGs in HCC.

## Conclusion

We developed a new necroptosis-related signature for predicting prognosis with the potential to predict immunotherapy for HCC patients.

## Data Availability

The datasets presented in this study can be found in online repositories. The names of the repository/repositories and accession number(s) can be found in the article/Supplementary Material.

## Abbreviations

CNV, Copy number variation; CDF, Cumulative distribution function; HPA, Human Protein Atlas; CLC, cluster consensus; ICGC, International Cancer Genome Consortium; LASSO, least absolute shrinkage, and selection operator; NRGs, necroptosis -related genes; ROC, Receiver operating characteristics; ssGSEA, single-sample gene set enrichment analysis; TCGA, The Cancer Genome Atlas; TIDE, Tumor Immune Dysfunction, and Exclusion; TMB, Tumor mutation burden.
