# Supplementary figures and images for: A necroptosis -related signature for predicting prognosis and immunotherapy in hepatocellular carcinoma
Source: Front Genet. 2022 Sep 5;13:919599. doi: 10.3389/fgene.2022.919599 (PMC9484537; doi:10.3389/fgene.2022.919599)

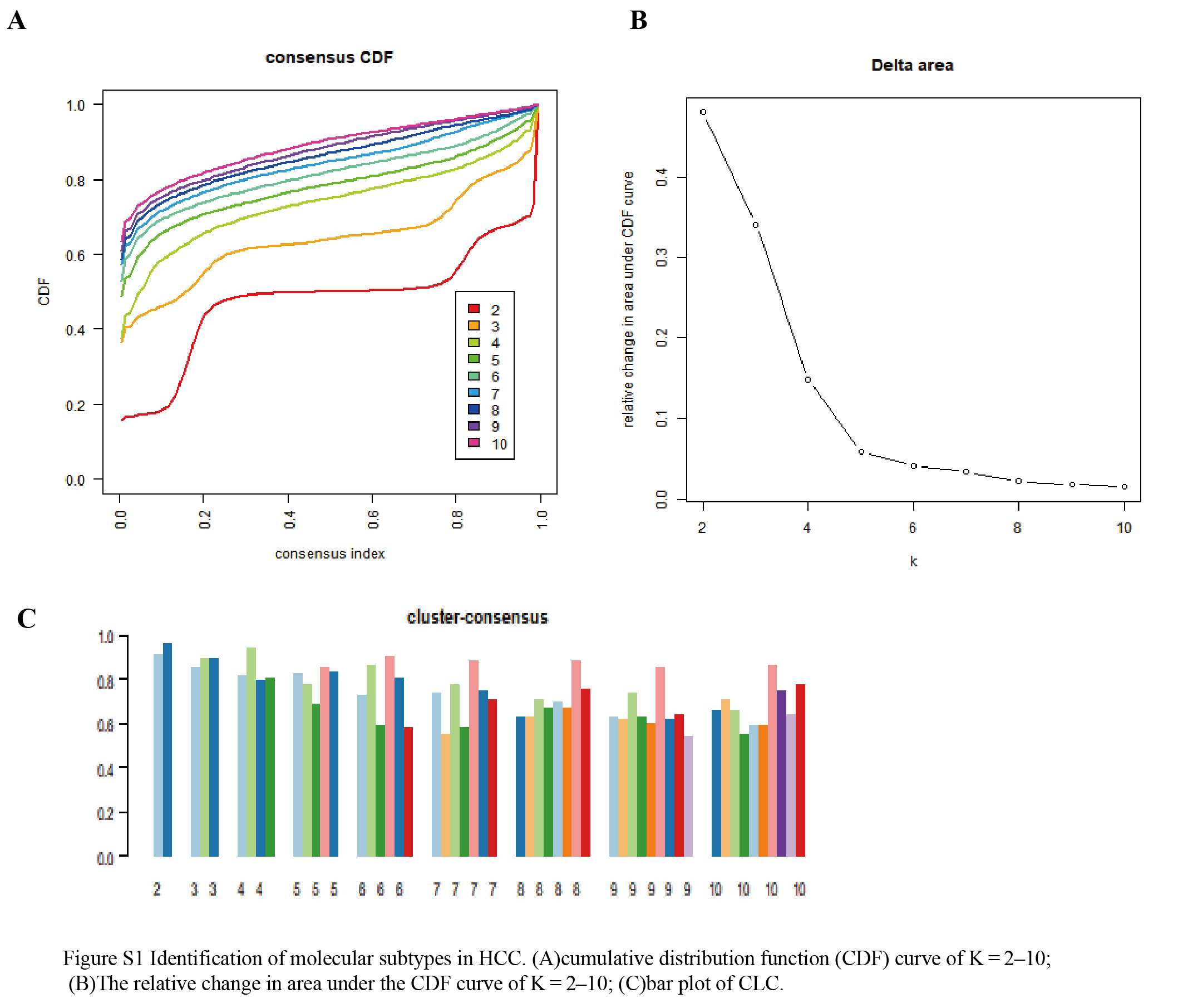

Supplement: Supplementary file 3 [file Presentation1.zip › Figure S1.JPEG]

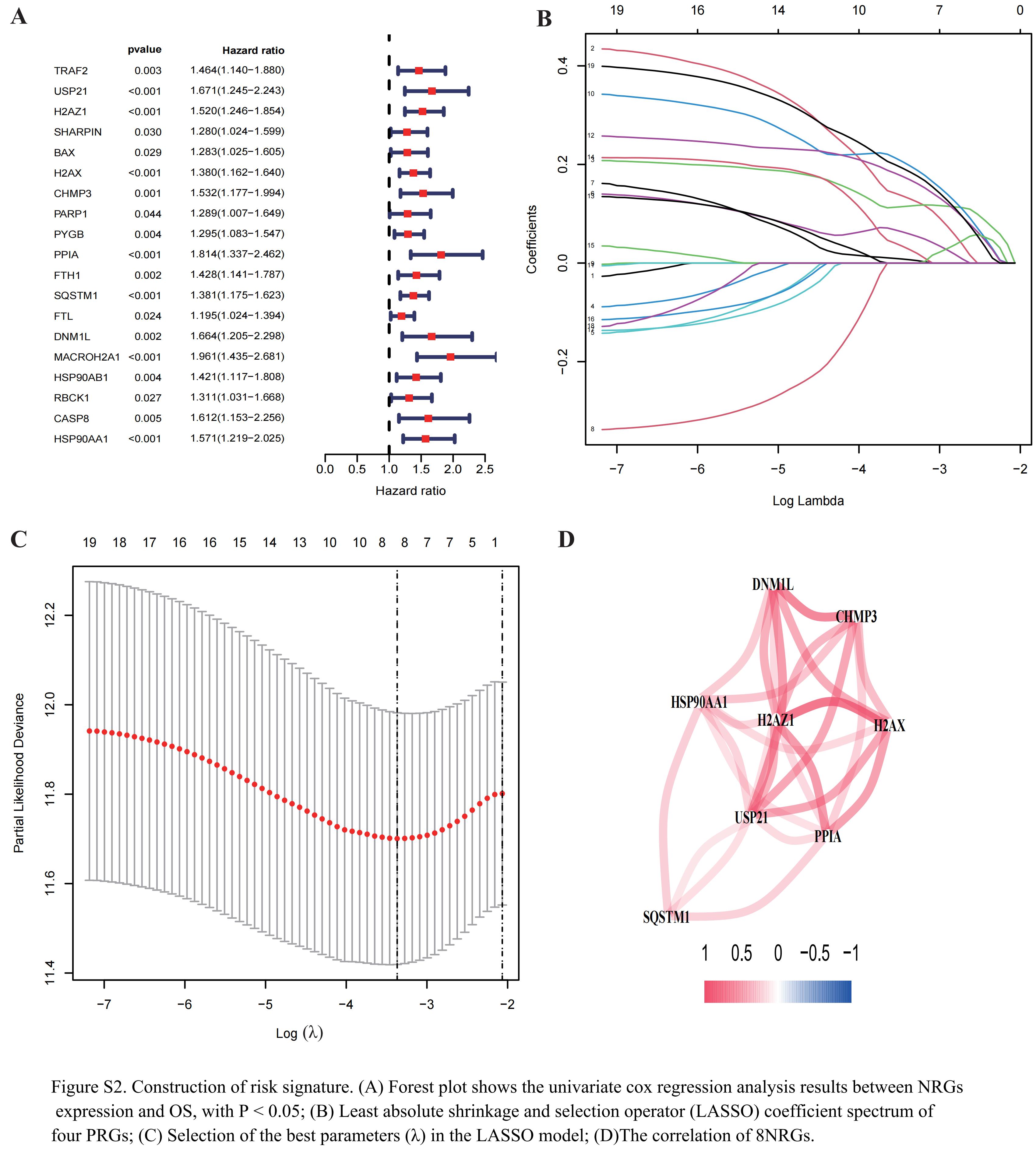

Supplement: Supplementary file 3 [file Presentation1.zip › Figure S2.JPEG]

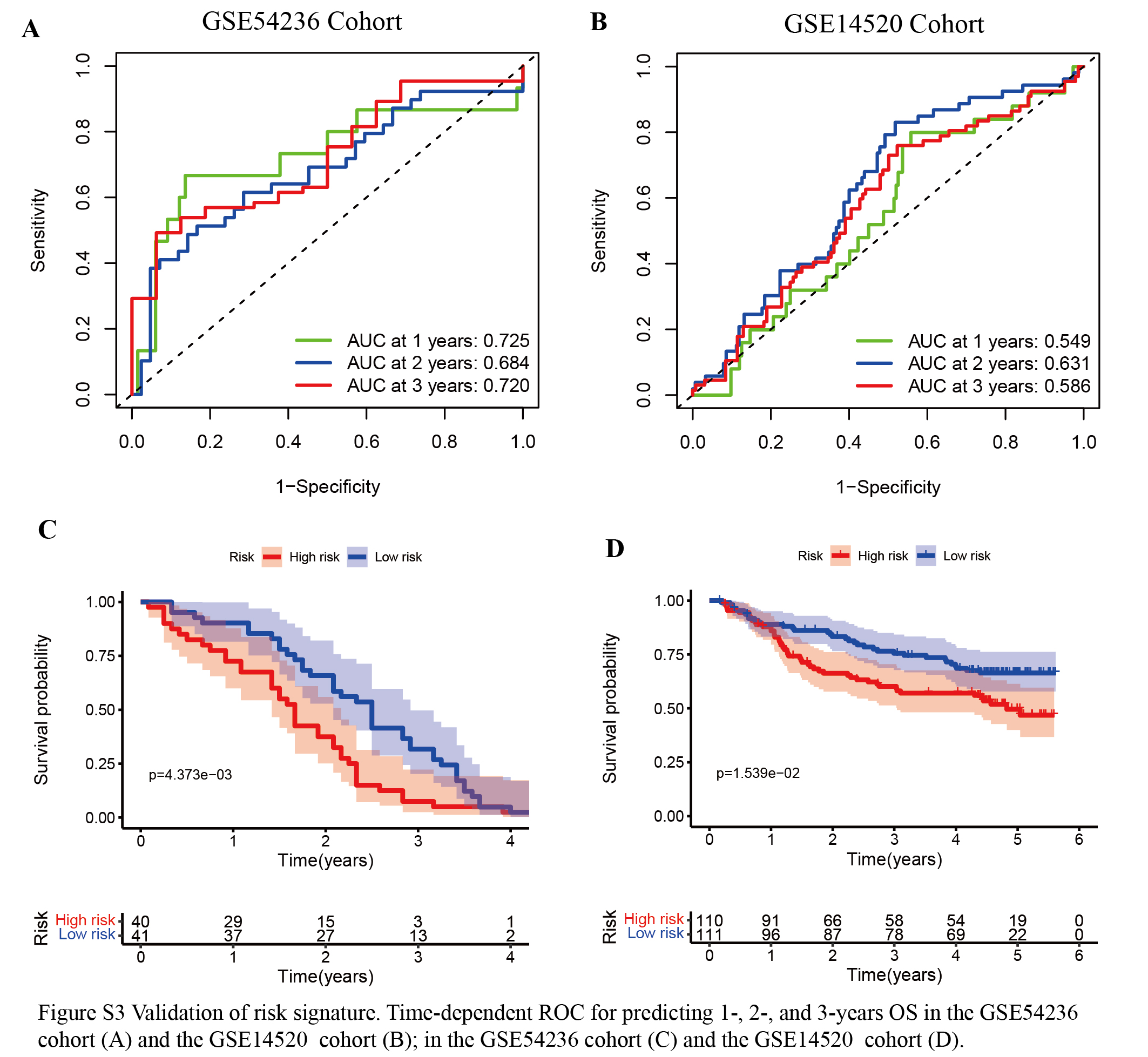

Supplement: Supplementary file 3 [file Presentation1.zip › Figure S3.JPEG]

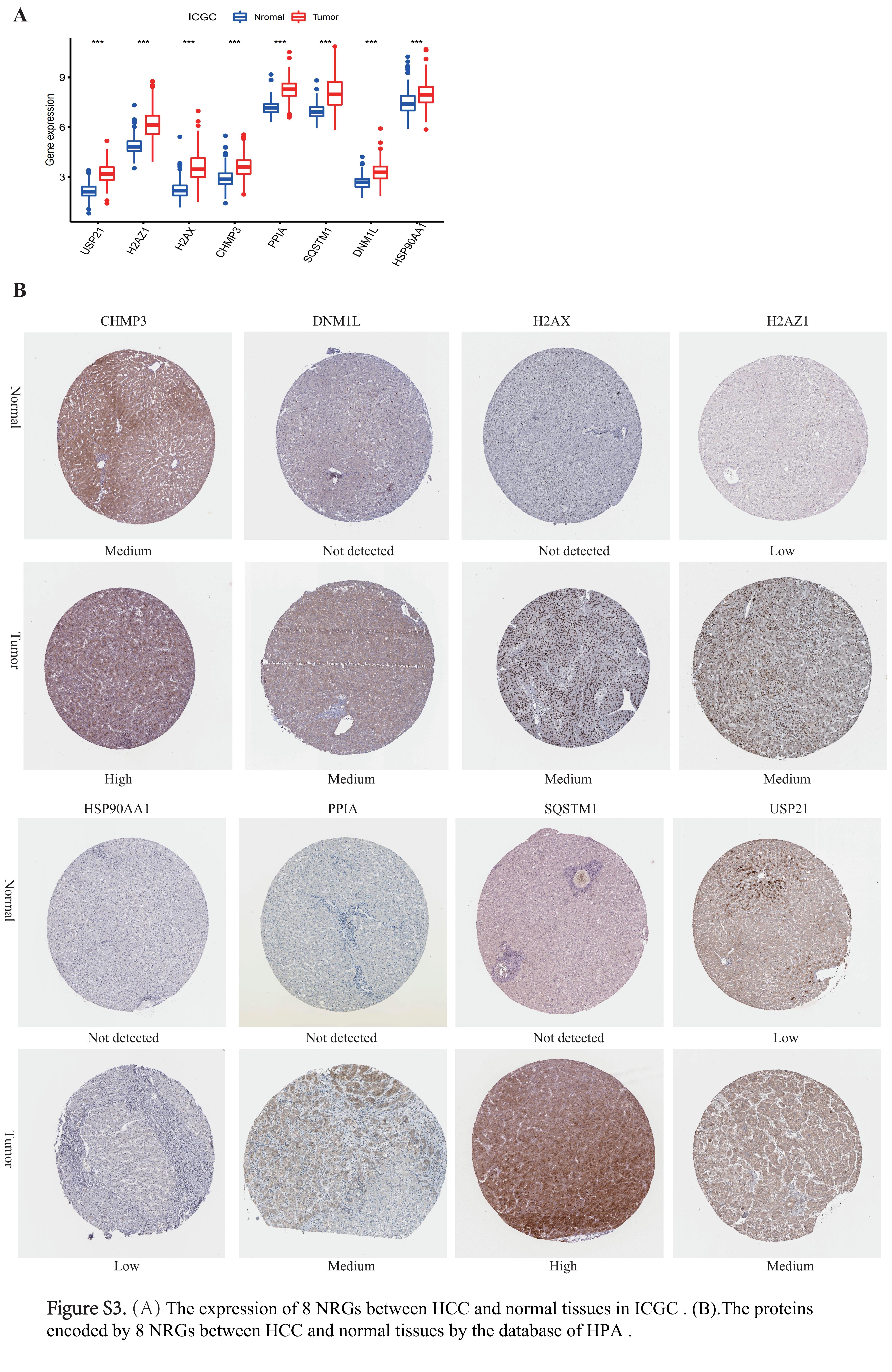

Supplement: Supplementary file 3 [file Presentation1.zip › Figure S4.JPEG]

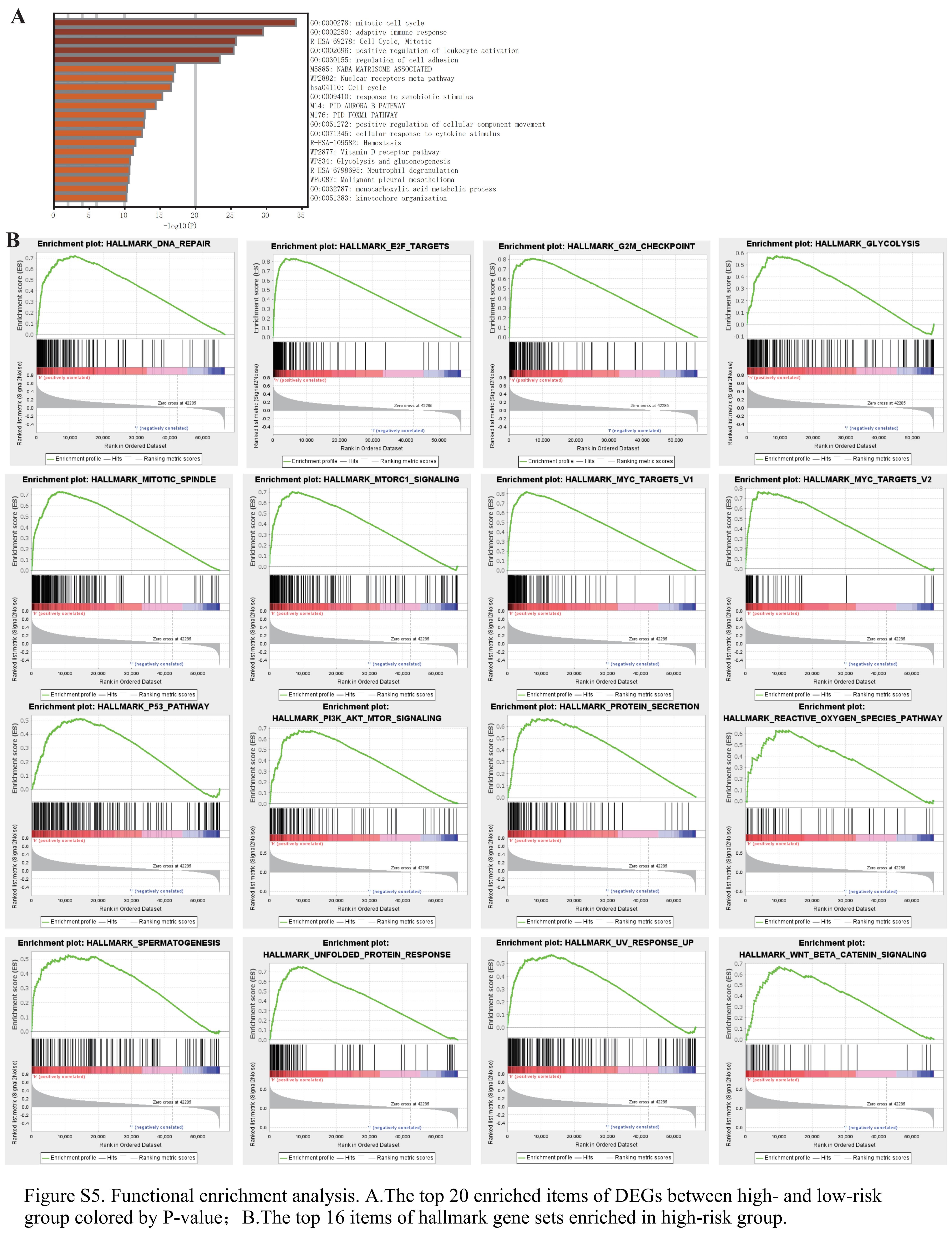

Supplement: Supplementary file 3 [file Presentation1.zip › Figure S5.JPEG]

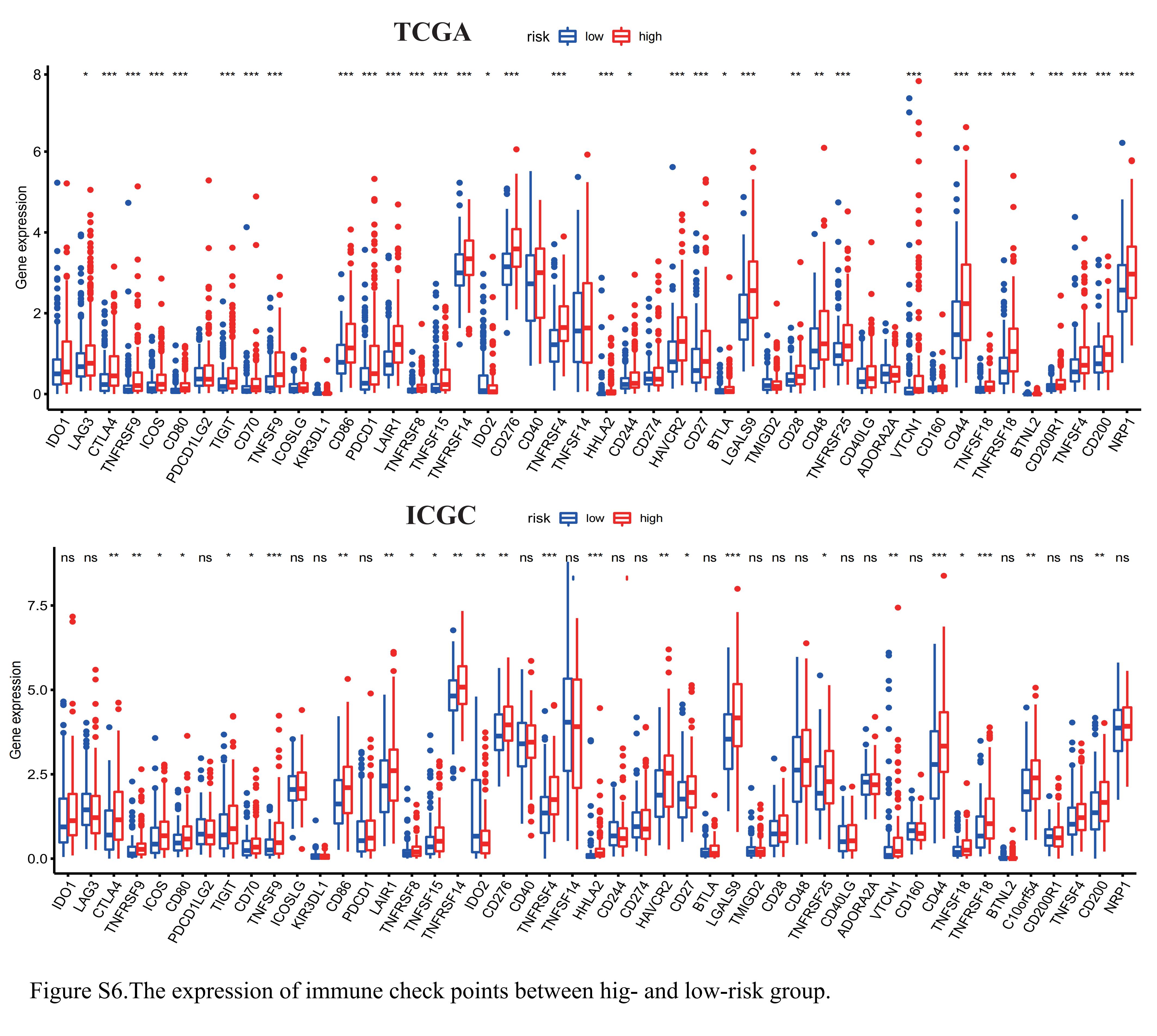

Supplement: Supplementary file 3 [file Presentation1.zip › Figure S6.JPEG]

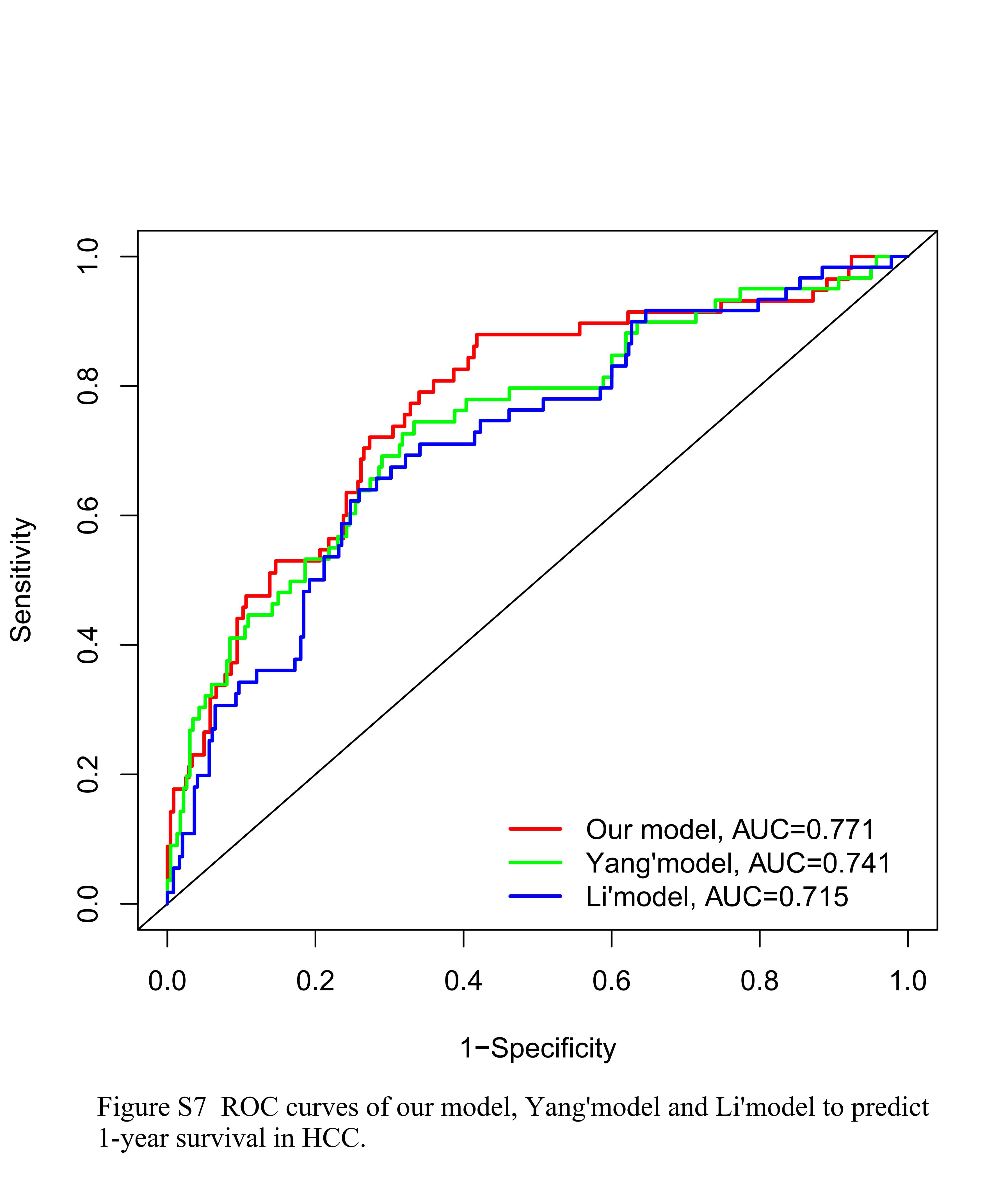

Supplement: Supplementary file 3 [file Presentation1.zip › Figure S7.JPEG]
